# Leveraging natural climatic advantages for large‑scale wheat doubled haploid production via wheat × maize: a protocol optimization study

**DOI:** 10.1186/s12870-026-09049-w

**Published:** 2026-05-26

**Authors:** Zhonghui Yang, Xiong Tang, Jian Yin, Sedhom Abdelkhalik, Shaoxiang Li, Kun Liu, Muhammad Asim, Armghan Shahzad, Ahmed Elfanah, Mingliang Ding, Mujun Yang, Hongsheng Li

**Affiliations:** 1 Food Crops Research Institute, Yunnan Academy of Agricultural Sciences, Kunming, 650205 China; 2 Wheat Research Department, Agricultural Research Center, Field Crops Research Institute, Giza, 12619 Egypt; 3 Plant Sciences Division, Pakistan Agricultural Research Council, Sector G-5/1, Islamabad, Pakistan; 4 National Institute for Genomics and Advanced Biotechnology, Pakistan Agricultural Research Council, Islamabad, 45500 Pakistan

**Keywords:** Wheat × maize, Doubled haploid, Flowering synchronization, Rolling pollination, Nutrient solution, Optical device, Caryopsis inspection, Yunnan

## Abstract

**Background:**

Doubled haploid (DH) lines generated via the wheat × maize system represent an effective technology for accelerating wheat breeding. However, achieving low-cost, large-scale DH production remains a core technical challenge.

**Results:**

In this study, we conducted flowering-synchronization experiments to determine the hybridization window between wheat and maize. Wheat was sown year-round in the field, while maize was planted both in the field and in high tunnels. We also evaluated a novel pollination method, four new nutrition solutions for cut-tiller culture, and a newly designed optical device for screening embryo-containing caryopses prior to embryo rescue. The results are as follows: (i) spring wheat, as well as vernalized facultative and winter wheat can be naturally grown year-round in Kunming, a plateau region in China with a mild climate. The hybridization window between wheat and maize is five months (June-October) in natural conditions, but can be extended to 10 months (April-next January) through high-tunnel maize cultivation in January-February and August-September; (ii) compared with commonly used brushing pollen pollination, the rolling pollination method increased pollination efficiency more than tenfold, and raised the haploid embryo formation frequency (EFF) by 1.9 times; (iii) one new nutrient solution (A) for cut-tiller culture performed significantly better than the currently used nutrient solution B. It achieved the highest EFF (46.39%), caryopsis setting rate (97.20%), and 1000-caryopsis weight (28.49 g) among five solutions. Solution A, without sulfurous acid and silver nitrate (both present in solution B), is also more cost-effective and environmentally friendly; (iv) pre-screening caryopses using a newly designed optical device prior to embryo rescue demonstrated a 98.6% accuracy rate and a 3.46% false-negative rate in selecting embryo-containing caryopses. This method doubles the efficiency of embryo rescue and remarkably reduces the use of sterilants and labor in mass DH production. The optimized protocols were validated from 2022 to 2024, yielding over 137,080 DH lines from 1,039 diverse wheat materials.

**Conclusions:**

The optimized protocol significantly improved doubled haploid production efficiency and reduced its cost, providing a more efficient and cost-effective system for mass production of wheat doubled haploids via wheat × maize. It is expected that the optimized protocol for wheat DH production will be applicable in regions with similar climates.

**Supplementary Information:**

The online version contains supplementary material available at 10.1186/s12870-026-09049-w.

## Background

Wheat is an indispensable crop that underpins global food security. To sustainably support food security, particularly amid ongoing climate change, developing climate-resilient cultivars is a critical priority [1]. Double haploid (DH) technology accelerates the breeding process and facilitates the release of resilient cultivars by rapidly developing fully homozygous lines in a single generation, thereby eliminating the need for multiple cycles of selfing and reducing dependence on empirical selection from segregating populations [2–4]. It has played a crucial role in modern crop breeding and genetic research for both cross- and self-pollinated crops such as maize, rapeseed, barley, and wheat [4–6]. Combining DH technology with marker-assisted selection or genomic selection, it unlocks unprecedented breeding efficiency [7–10]. Moreover, haploid embryos, which contain a single set of chromosomes, reduce genomic complexity and thus facilitate high-throughput gene editing and functional genomics research [10].

In wheat, three approaches stand out as the most robust and versatile strategies for doubled haploid production: anther or isolated microspore culture, a well-established in vitro technique [2, 11, 12]; chromosome elimination through wide cross, a classic method leveraging interspecies genetic incompatibility [13]; and gene-edited haploid inducer lines, a modern biotechnological innovation [7, 14]. Among them, the wide cross between wheat and maize, followed by maize chromosome elimination, represents the most effective and widely adopted method for producing DH lines in wheat breeding, featuring less genotype dependence, a simple workflow, and low infrastructure requirements [15–18]. Decades of research has established a widely implemented protocol for this DH system [17], including emasculation of wheat spikes [19, 20], pollination of emasculated spikes with maize pollen [21–23], hormone treatment [24–26], haploid embryo induction and development by in vivo or in vitro culture [27–29], embryo rescue and germination into haploid seedlings [30], chromosome doubling treatment of haploid seedlings [17, 31, 32], transplantation of treated plants [33, 34], and harvest of DH lines [8, 18].

To enable the wide application of DH technology in wheat breeding, mass production of DH at low cost is essential, which requires an efficient protocol. Although the DH protocol based on wide cross between wheat and maize has been continuously optimized [17, 18, 29, 35, 36], some challenges in large-scale production still exist and constrain improvements in DH production efficiency and cost reduction: (1) wheat and maize are typically planted in different seasons in most regions of the world, requiring controlled conditions for synchronizing their flowering times, which substantially increases the costs of DH production [37]. However, potential solutions may exist in South Asia, Africa, and high-altitude regions [38]; (2) pollinating emasculated wheat spikes with maize pollen on a floret-by-floret way is labor-intensive and time-consuming, which may also negatively affect the haploid embryo induction efficiency, as maize pollen retains high viability for only a limited duration [29, 34, 38, 39]; (3) generally less than one-third of the thousands of caryopses generated from wheat × maize contain haploid embryos [2, 39]; however, the present embryo rescue procedures necessitate the sterilization and dissection of all caryopses [17], resulting in substantial waste of reagents, labor, and time on embryo-free caryopses, thereby reducing the operational efficiency and significantly increasing production costs. Additionally, in vitro culture of pollinated wheat tillers has been proven to be an efficient method for maintaining stable and high haploid embryo formation frequency [27, 29, 35, 40]. However, a more cost-effective and environment-friendly nutrient solution for cut-tiller culture remains to be developed.

To address challenges above, over the past ten years, we have been committed to developing a high-throughput protocol to produce DH for accelerating wheat breeding by leveraging the advantageous climate of Kunming, Yunnan province [39, 41], a plateau region in southwestern China where spring and vernalized winter wheat can be grown year-round, facilitating synchronization of wheat-maize flowering periods. Based on previous researches in DH production [29, 33, 34, 38, 39], this study aimed to (i) determine and expand the crossing window between wheat and maize, (ii) evaluate the efficiency of a novel pollination method, (iii) optimize the nutrient solution for cut-tiller culture and haploid embryo induction, and (iv) assess the feasibility of using the new optical device for screening caryopses with embryos prior to embryo rescue. Collectively, these efforts aim to establish a large-scale, cost-effective DH production protocol.

## Materials and methods

Wheat materials used in this study were obtained from the breeding programs at different institutions in China, Egypt, and Pakistan. All experiments were conducted at the wheat DH production base or DH laboratory of Yunnan Academy of Agricultural Sciences (YAAS), Kunming, Yunnan province, China.

The complete DH production protocol and workflow adopted in this study are outlined as follows: (1) plant wheat and maize materials in multiple sowing dates; (2) cut the emasculated wheat tillers at ground level within 24–48 h after emasculation, pollinate with fresh maize pollen using the rolling pollination method [29], and place them into nutrient solution; (3) spray spikes with 100 mg/L 2,4-dichlorophenoxy acetic acid (2,4-D) solution 24 h after pollination, and then culture tillers in a growth chamber for 14 days, under a regime of 14 h light/10 h darkness, and 8000 lx light intensity, constant temperature of 23 ± 1℃, and 80 ± 5% relative humidity. The nutrient solution was replaced every three days [17, 29, 38, 39]; (4) select caryopses containing embryos and aseptically dissect them for embryo rescue with half-strength MS medium as routine procedures [38]; (5) when seedlings germinated from embryos develop tillers, conduct chromosome doubling with 0.06% colchicine solution for 5 h, followed by washing seedlings for 3 h with tap water; (6) transplant seedlings into trays containing substrate for one month, then transplant the survived seedlings to the field.

### Determination of the crossing duration between wheat and maize

Fifteen spring, facultative and winter wheat cultivars in China (Table 1) and one maize inbred line ‘Bai-Tian-Nuo’ (developed by YAAS) were used in this experiment to determine the crossing duration between wheat and maize. For spring wheat, 52 sowing dates at weekly intervals were evaluated over one year (starting on January 1, 2020) using seven spring bread wheat genotypes. Days to heading (DTH) was recorded as the duration from sowing to 50% heading per plot.

**Table 1:** Fifteen typical spring, facultative and winter wheat cultivars

Cultivars	Pedigree	Type	Cultivars	Pedigree	Type
02Y11-18	Advanced lines	Spring	Huaimai 22	Huaimai 18/Yangmai 158	Facultative
02Y11-25	Advanced lines	Spring	Zhoumai 22	Zhoumai 12/Wenmai 6//Zhoumai 13	Facultative
02Y11-6	Advanced lines	Spring	Wanmai 52	Zhengmai 8329/Wanmai 19	Facultative
K1564S	96B-138/C49S	Spring	Huaimai 25	Dwarf male-sterile wheat recurrent selection population	Facultative
K74S	K78S/K456S	Spring	Jingdong 17	Jingdong 8/RHT 3//931	Winter
K78S	96B-138/C49S	Spring	Lunxuan 518	Dwarf male-sterile wheat recurrent selection population	Winter
K80S	K78S/K456S	Spring	Jingdong 22	Dwarf male-sterile wheat recurrent selection population	Winter
			Jinghua 9	Jingshi 467/Jingdong 8/Jingdan 93-2197	Winter

According to our previous experiments, facultative and winter wheat were evaluated under two cultivation regimes: (i) direct sowing (without vernalization) in winter and spring, and (ii) summer sowing after vernalization. Four facultative and four winter wheat genotypes (without vernalization) were arranged in a randomized complete block design (RCBD) with three replicates; each plot consisted of five 1 m rows spaced 0.30 m apart. Direct sowing was conducted on February 7, 2020, and the summer sowing (after vernalization) on May 25, 2020. DTH was recorded as above.

Field sowing of maize: generally, maize can be sown in open fields at Kunming from March to July, while maize pollen is typically available from June to October. In this experiment, the inbred line ‘Bai-Tian-Nuo’ was sown at 14-day intervals from March 4 to July 22 in 2020 and from March 1 to July 19 in 2021 (Table 2). Off-season pollen supply via high tunnel cultivation: To ensure a year-round supply of maize pollen for the year-round planting of wheat, maize was additionally cultivated in the high tunnel at 14-day intervals during the 2020 and 2021 seasons (Table 2). The flowering dates of each sowing were recorded.

**Table 2:** The eleven sowing times in the field and nine maize sowing dates in the high tunnel during the 2020 and 2021 seasons

Field environment		High tunnel environment	
Sowing date (2020)	Sowing date (2021)	Sowing date (2020)	Sowing date (2021)
4-Mar	1-Mar	14-Jan	1-Jan
18-Mar	15-Mar	28-Jan	15-Jan
1-Apr	29-Mar	9-Feb	29-Jan
15-Apr	12-Apr	23-Feb	12-Feb
29-Apr	26-Apr	5-Aug	1-Aug
13-May	10-May	19-Aug	15-Aug
27-May	24-May	2-Sep	29-Aug
10-Jun	7-Jun	16-Sep	12-Sep
24-Jun	21-Jun	30-Sep	26-Sep
8-Jul	5-Jul		
22-Jul	19-Jul		

### Evaluation of the novel pollination method (rolling pollination)

**Fig. 1:**
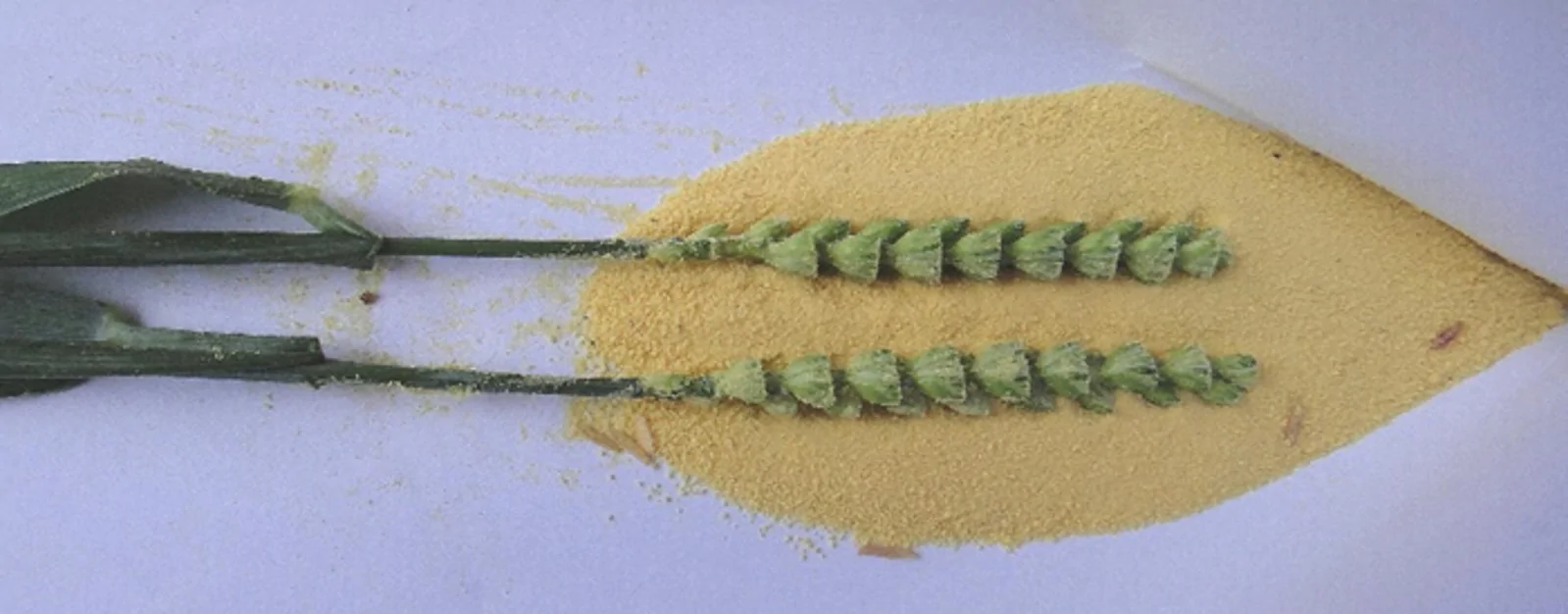
Illustration of rolling pollination

Rolling pollination: fresh maize pollen was collected from 10:00 to 11:30 a.m. After filtering or removing the anthers, the pollen was kept on dry paper. Then one or two emasculated wheat spikes (at feathery stage) were inserted into the pollen, followed by slightly rolling the spike or shaking the paper. Each pollination was completed within 2 s (Fig. 1).

To compare the efficiency of rolling pollination with the traditional brushing pollen pollination method [18], a comparative experiment was conducted in 2022. Emasculated wheat spikes from the same cultivar ‘Yunmai 112’ (developed by YAAS) were pollinated with maize pollen from ‘Bai-Tian-Nuo’ using rolling pollination and brushing pollen pollination, respectively. For brushing pollen pollination, a fine brush was used to apply maize pollen to the stigma of individual florets. The experiment was conducted by the same person on three different days (as three replications). Pollination efficiency (PE, pollinated spikes per hour by the same person) of each method was recorded. All pollinated spikes were in vitro cultured following the DH production protocol described above. After 14 days of culture [29], caryopses were collected to count the caryopsis setting rate (CSR), embryo formation frequency (EFF), and selfing rate (SR). The parameters were calculated using the following formulas:

$$\begin{array}{l}{\mathrm{PE}=\left(\text{Number of pollinated spikes}\right)/\left(\text{Pollination time in hours}\right)}\\{\mathrm{CSR} \left({\%}\right)=\left(\text{Total number of caryopses}\right)/\left(\text{Total number of pollinated florets}\right)\times\mathrm{100}}\\{\mathrm{EFF}\left({\%}\right)=\left(\text{Total number of embryos}\right)/\left(\text{Total number of caryopses}\right)\times\mathrm{100}}\\{\mathrm{SR}\left({\%}\right)=\mathrm{100-}\mathrm{CSR}}\end{array}$$

### Nutrient solution experiment

Four newly designed nutrient solutions (A, C, D, E; Table 3) and the currently used solution B (control) for cut-tiller culture were applied to compare their effects on haploid induction. 24 h after pollination with ‘Bai-Tian-Nuo’, the wheat tillers from the same cultivar ‘Yunmai 112’ were cut for in vitro culture with five nutrient solutions according to the protocol described above. The experiment was conducted in the summer sowing of 2022, with three replicates of approximately 50 spikes per treatment. After 14 days of culture, twelve spikes from each treatment were randomly collected to count the total pollinated florets, total number of caryopses, number of caryopses containing embryos, 1000-caryopsis weight (CW), caryopsis setting rate (CSR), and embryo formation frequency (EFF). CW was calculated using the following formula:

$$\mathrm{CW}=\left(\text{Total weight of caryopses}\right)/\left(\text{Total number of caryopses}\right)\times\mathrm{1000}$$

**Table 3:** Five nutrient solutions (A, B, C, D, and E) and their components

Components	A	B^*^	C	D	E
2,4-Dichlorophenoxyacetic Acid (2,4-D) (mg/L)	100	100	100	100	100
Sucrose (C_12_H_22_O_11_) (mg/L)	40	40	40	40	40
Potassium Dihydrogen Phosphate (KH_2_PO_4_) (g/L)	4	4	4	4	4
Sulfurous Acid (H_2_SO_3_) (mL/L)	-	8	-	-	-
Sodium Tetraborate Decahydrate (Na_2_B_4_O_7_) (g/L)	1.5	1.5	1.5	-	1.5
Silver Nitrate (AgNO_3_) (mg/L)	-	8	8	-	-
Boric Acid (H_3_BO_3_) (mg/L)	100	-	-	100	100
Citric Acid (C₆H₈O₇) (mg/L)	20	-	-	20	20
Sodium Bisulfite (NaHSO₃) (mg/L)	-	-	0.8	0.8	-
Brassinolide (C_28_H_48_O_6_ ) (mg/L)					0.2

* a currently-used solution, as control here; ‘-’ indicates the component is absent

### Evaluating a novel method of pre-screening embryo-containing caryopses for embryo rescue using an optical device

**Fig. 2:**
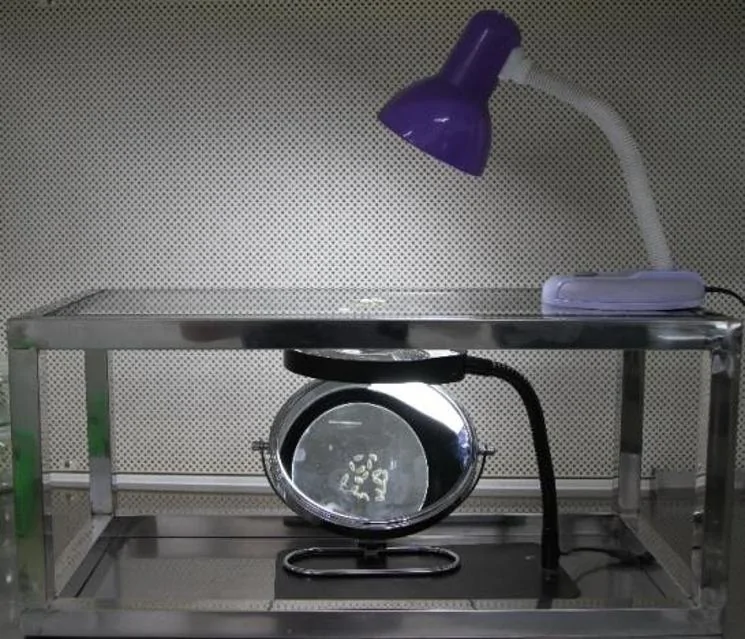
Schematic diagram of caryopses detection with an optical device

We designed an optical device to select the embryo-containing caryopses prior to embryo rescue (Fig. 2). In the summer sowing of 2022, 1,000 caryopses were randomly divided into two groups, with 500 caryopses in each group. One group underwent embryo rescue following routine procedures (C1), including caryopsis sterilization, aseptic dissection of caryopses and embryo inoculation onto the medium; another group was first screened using the optical device to select embryo-containing caryopses, which were then subjected to the same embryo rescue procedures (C2). To ensure detection accuracy in C2, the remaining caryopses were turned over and shaken after each screening round, and the screening was repeated three more times to recover any missed embryo-containing caryopses.The same person repeated the experiment three times, recording the times to complete C1 and C2 for each repeat, and the difference in embryo counts obtained by device detection versus manual dissection in C2.

To assess the reliability of the optical detection method (C2), the accuracy, false positive rate (FPR), and false negative rate (FNR) were calculated from a confusion matrix generated by comparison with method C1, using the following definitions: true positive (TP) denotes caryopses correctly identified as containing embryos; true negative (TN) denotes caryopses correctly identified as lacking embryos; false positive (FP) denotes caryopses without embryos incorrectly identified as containing embryos; and false negative (FN) denotes caryopses with embryos incorrectly identified as lacking embryos. Based on these definitions, accuracy, FPR, and FNR were calculated using the following formulas:

$$\begin{array}{l}{\mathrm{Accuracy}\left({\%}\right)=\left(\mathrm{TP+TN}\right)/\left(\mathrm{TP+TN+FP+FN}\right)\times\mathrm{100}}\\{\mathrm{FPR}\left({\%}\right)=\mathrm{FP/}\left(\mathrm{TN+FP}\right)\times\mathrm{100}}\\{\mathrm{FNR}\left({\%}\right)=\mathrm{FN}/\left(\mathrm{TP+FN}\right)\times\mathrm{100}}\end{array}$$

### Statistical analyses

Data were collected and analyzed using Microsoft Excel 2016. Bar and line charts were created using Python version 3.x (Python Software Foundation, Beaverton, OR, USA) with the Matplotlib library.

## Results

### The annual crossing window for wheat × maize is five months under field conditions, and can be extended to 10 months with the high tunnel

**Fig. 3:**
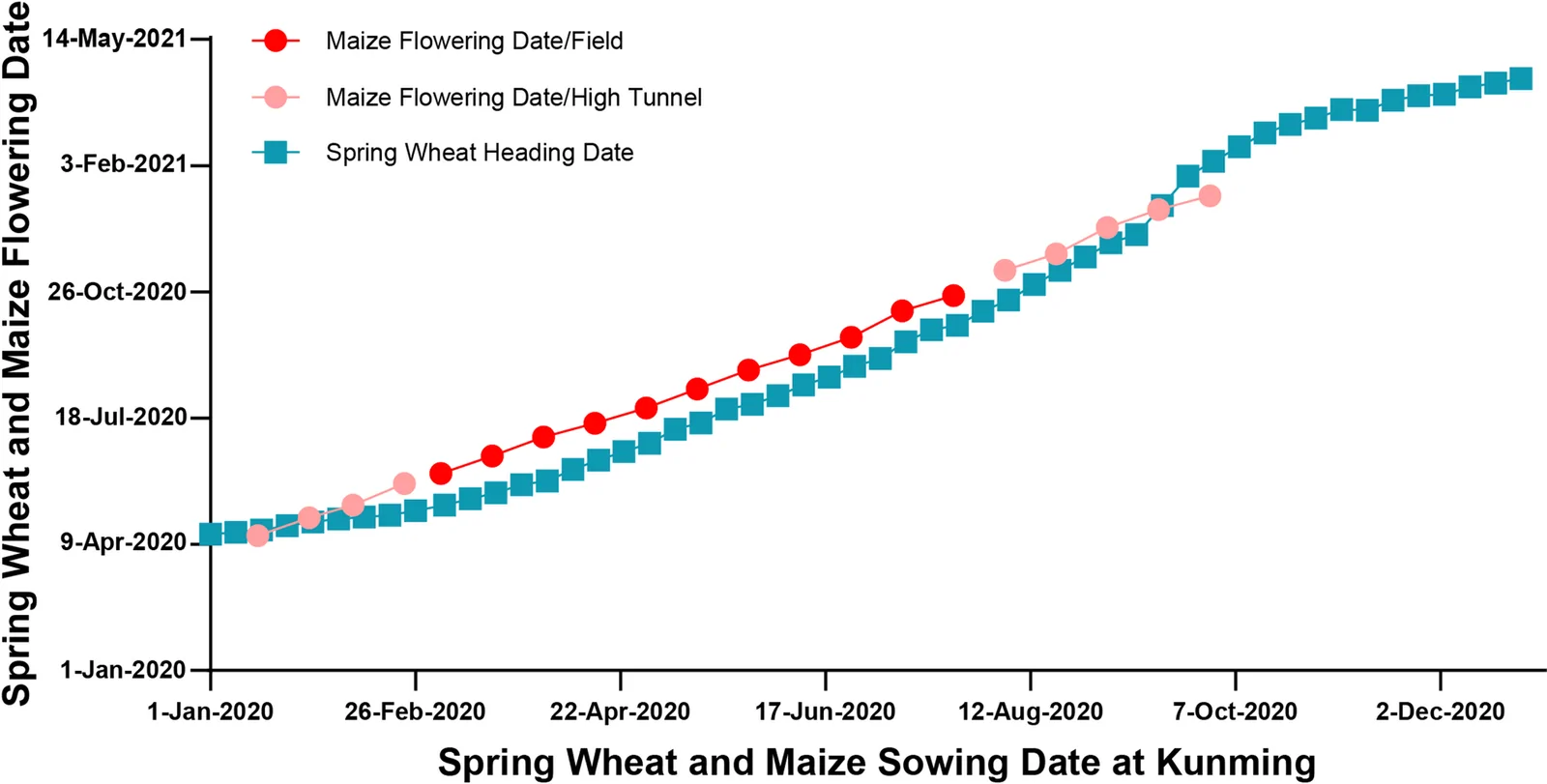
Flowering synchronization of spring wheat and maize under field conditions during the 2020 cropping season

Figure 3 illustrates the heading dates of seven spring wheat cultivars across 52 field sowing dates, and the flowering periods of maize in 20 sowing dates during the 2020 cropping season. Results demonstrated that the spring wheat exhibited normal heading across all 52 sowing dates, with heading periods spanning from January to December, whereas maize exhibited flowering from June to October, suggesting the crossing window for DH production is five months from June to October under the field conditions at Kunming. The shortage of maize pollen supply limited the DH production during the remaining months.

**Fig. 4:**
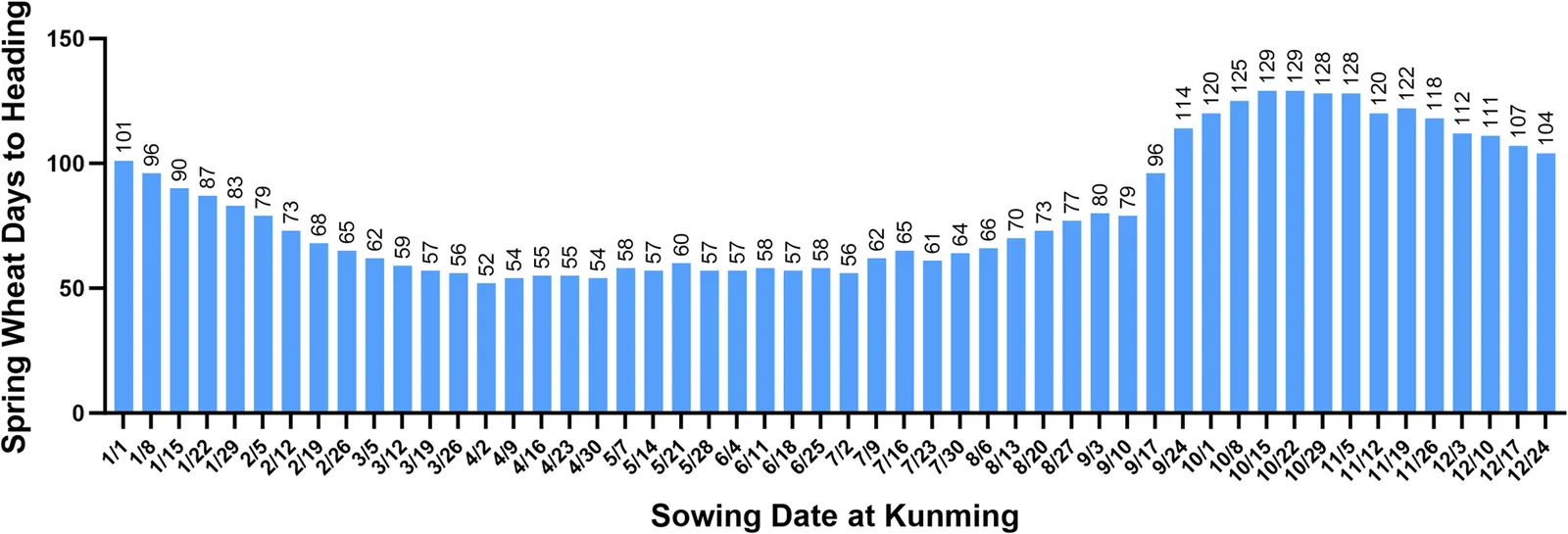
Days to heading of seven spring wheat cultivars across 52 sowing dates (7-day intervals) in 2020

Although spring wheat can be naturally sown year-round at Kunming, the DTH (days to heading, *P* ≤ 0.001) showed significant differences among 52 sowing dates, ranging from 52 ± 1.1 days (sown on April 2, 2020) to 129 ± 1.3 days (sown on October 22, 2020). No significant differences in DTH were observed among the seven wheat cultivars (*P* = 0.893 ). Sowing spring wheat from late February to early August appears more cost-effective for DH production due to the shorter DTH (52–67 days), and their heading times (from June to October) also align well with the maize flowering times (Fig. 4, Table S1).

**Fig. 5:**
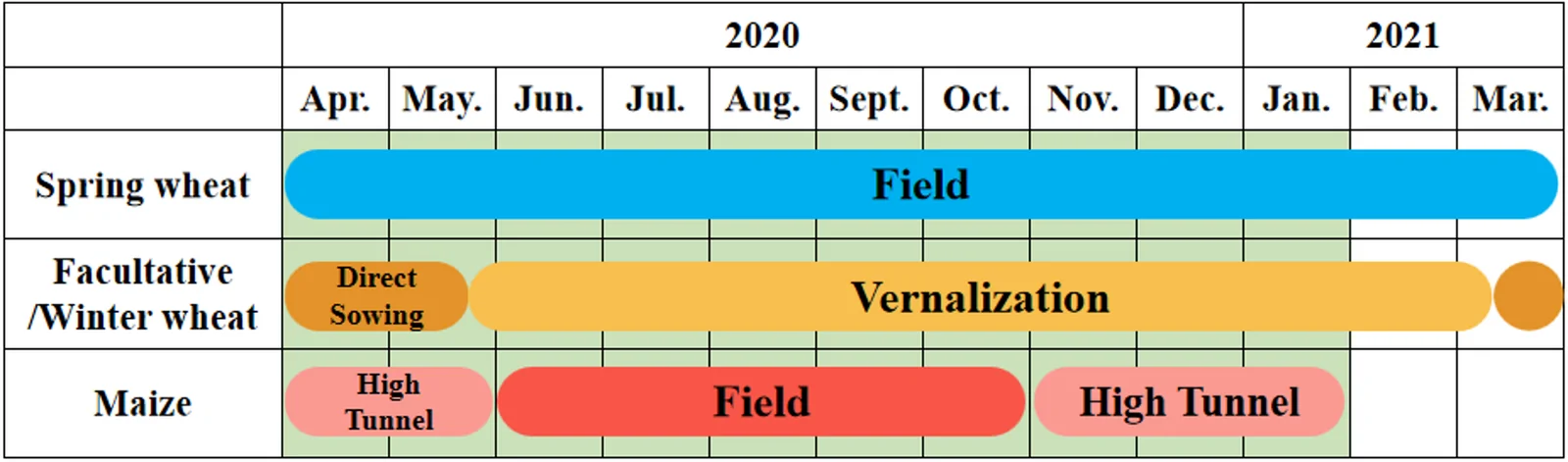
Flowering synchronization of spring wheat, facultative/winter wheat (with and without vernalization), and maize (field and high tunnel) in 2020–2021. Horizontal bars: flowering/heading periods. Green shading: optimal DH production window. X-axis: April 2020 -March 2021

Generally, facultative and winter wheat from northern China can not head normally during summer sowing at Kunming in the absence of vernalization treatment; however, when sown in early February, they exhibited normal heading in May, matching the flowering periods of high tunnel-grown maize. In contrast, the vernalized facultative and winter wheat sown in early May at Kunming headed well, the heading time (in August) also coincided with the flowering periods of field-grown maize (Fig. 5).

As a supplementary strategy to extend the pollen supply, maize was planted in a high tunnel during January-February and August-September in 2020 and 2021. In both years, the high tunnel flowering windows occurred consistently from April to May and from November to January (Table S2, Fig. 5). These results, combined with the flowering window of field-grown maize (June to October), ensure 10-month availability of maize pollen for DH production (Fig. 5), which also enhances the flexibility of sowing time when using facultative and winter wheat to produce double haploids.

### Rolling pollination: a transformative approach to boosting pollination efficiency

As shown in Table 4, significant differences were observed between the traditional brush pollination method and the rolling pollination method in embryo formation frequency (EFF) and pollination efficiency (PE). The EFF of the rolling pollination method was 31.12% ± 4.60%, approximately 2.9-fold higher than that of the traditional method (10.62% ± 1.49%, *P* = 0.0018). In terms of pollination efficiency, the rolling pollination method achieved an average of 643.33 ± 49.33 spikes per hour, an 11.8-fold increase over the traditional method (54.67 ± 7.33 spikes per hour; *P* < 0.0001). Notably, no significant differences were observed between the two methods in caryopsis setting rate (CSR) and selfing rate (SR). These parameters primarily reflect emasculation thoroughness rather than the pollination method, indicating consistent emasculation quality across treatments. Collectively, these results demonstrate that the rolling pollination method significantly improves both embryo formation frequency and operational efficiency compared with the traditional brush pollination method.

**Table 4:** Comparison of pollination efficiency and embryo formation frequency between traditional brush pollination and rolling pollination methods

Parameter	Traditional brush pollination	Rolling pollination	*P-*value	Significance
No. of pollinated spikes	100	100	—	—
No. of pollinated florets	2817.0 ± 414.4	2853.3 ± 356.5	—	—
No. of haploid embryos	275.7 ± 22.8	827.7 ± 43.4	—	—
EFF(%)	10.62 ± 1.49	31.12 ± 4.60	0.0018	**
PE (spikes/h)	54.67 ± 7.33	643.33 ± 49.33	< 0.0001	***
CSR (%)	93.22 ± 2.22	94.30 ± 1.45	0.512	ns
SR (%)	6.78 ± 2.22	5.70 ± 1.45	0.512	ns

Data are presented as mean ± standard deviation (*n* = 3 replicates). *P* values were calculated using the independent samples Student’s t-test. ** *P* < 0.01, *** *P* < 0.001, ns: not significant (*P* > 0.05)

**Fig. 6:**
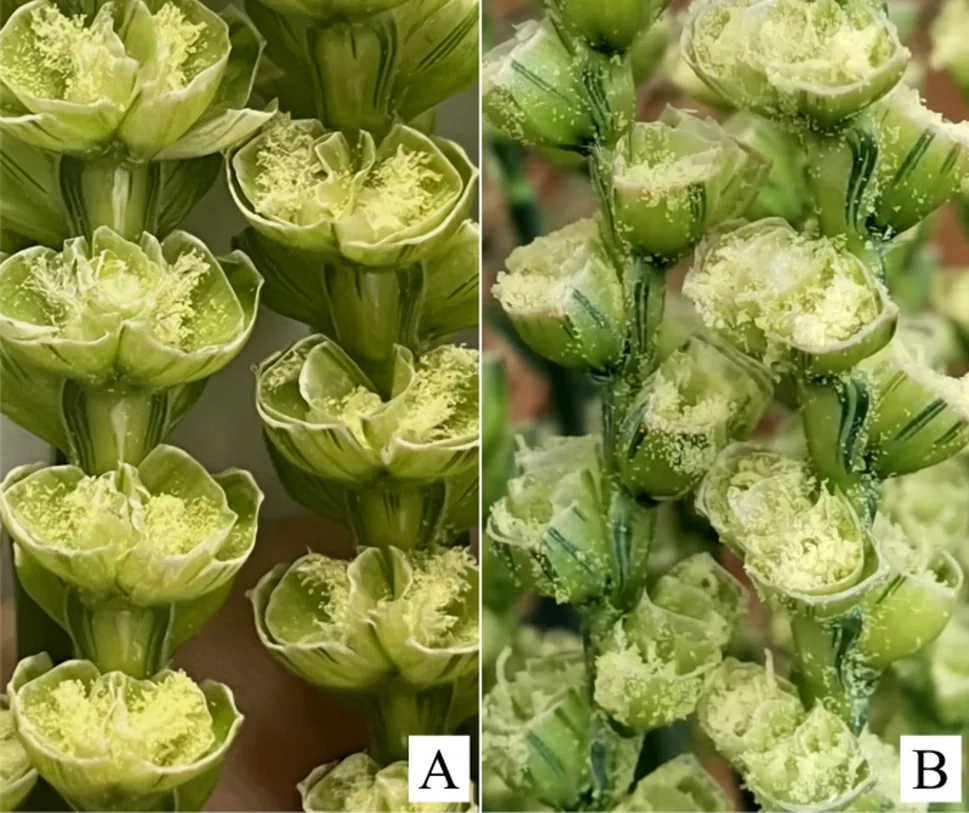
Post-pollination comparison of wheat spikes pollinated with the traditional brush method (**A**) and the rolling pollination method (**B**), showing differences in pollen coverage

After pollination, maize pollen covered almost the entire stigma surface, eliminating the need for bagging (Fig. 6B).

### Optimized nutrient solution exhibited reduced cost, enhanced embryo induction efficiency, and improved environmental performance

The performance of five nutrient solutions for cut-tiller or detached tiller culture in haploid induction is summarized in Table 5. We found that solution A had the highest 1000-caryopsis weight (CW) at 28.49 g, which was significantly higher than that of the other tested nutrient solutions; meanwhile, it also exhibited significantly higher caryopsis setting rate (97.20%) and embryo formation frequency (46.39%) than the currently used nutrient solution B (control). Additionally, in solution A, sulfurous acid (H_2_SO_3_), a corrosive and irritating substance, and silver nitrate(AgNO_3_), a costly, corrosive, and hazardous chemical, have been removed compared with solution B. These results indicate that solution A has lower cost, higher embryo induction efficiency, and better environmental friendliness.

**Table 5:** Mean performance of five nutrient solutions applied for DH production in 2022

Solution	1000-caryopsis weight CW (g)	Caryopsis setting rate CSR (%)	Embryo formation frequency EFF (%)
A	28.49^†a^	97.20^a^	46.39^a^
E	24.96^b^	94.11^ab^	38.25^ab^
D	22.54^bc^	93.86^ab^	35.20^b^
B (control)	21.92^c^	92.90^b^	32.03^b^
C	20.27^c^	91.74^b^	28.97^b^
Significance	**	**	**
CV%	4.5	1.7	11.9

^†^Mean values within the same column for each trait with the same lower-case letter are not significantly different according to Fisher’s least significant difference (FLSD) test at *P* ≤ 0.01. ** *P* < 0.01

**Fig. 7:**
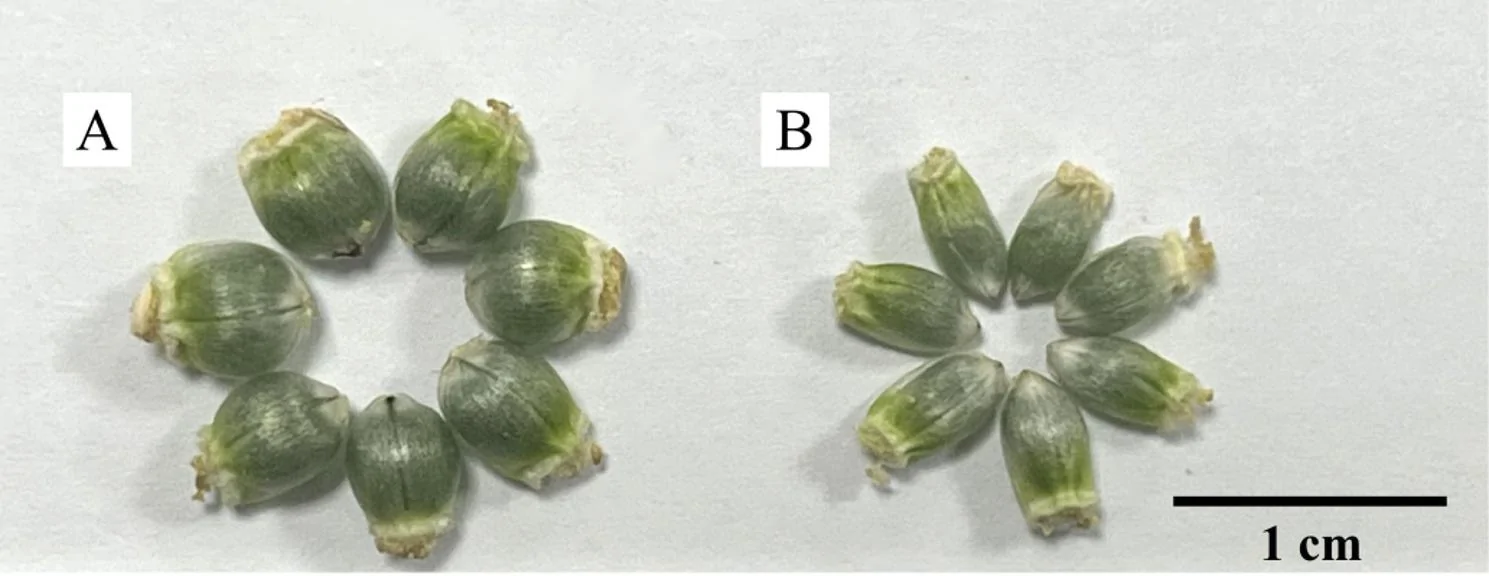
Caryopses developed from solution **A** and **B**

In addition, larger and rounder caryopses from solution A are also more advantageous in the subsequent caryopsis detection using an optical device to select embryo-containing caryopses for embryo rescue (Fig. 7).

### The application of an optical device in embryo rescue remarkably enhanced operating efficiency, with only slight embryo loss

**Fig. 8:**
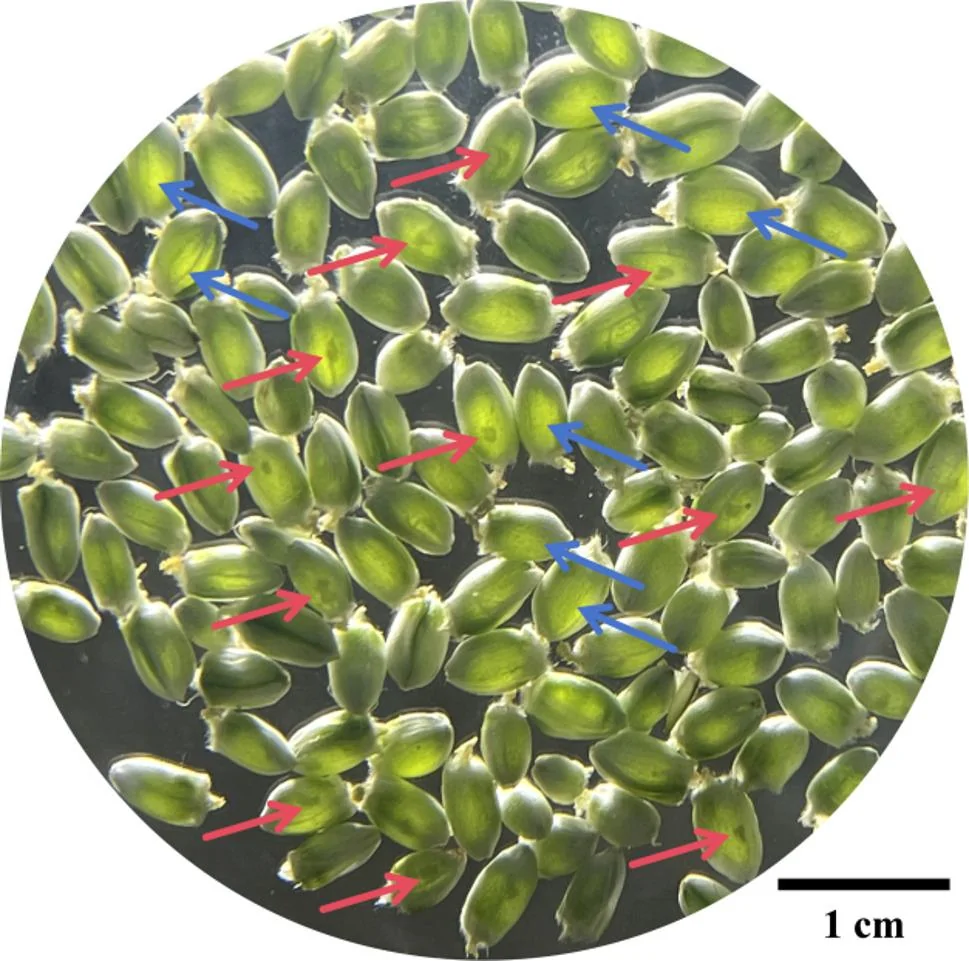
Results of a caryopsis screening using the optical device. Red arrows indicate embryo-containing caryopses; blue arrows indicate the embryo-free caryopses

The effects of pre-screening embryo-containing caryopses with an optical device before conducting routine embryo rescue are shown in Table 6, and Fig. 8 illustrates the results of caryopsis screening using the optical device, which was repeated three times in this study. The routine method C1 achieved 100% accuracy with no false positives and false negatives, serving as the control. C1 was significantly more accurate than C2 (*P* = 0.0067), with lower false positive and false negative rates (*P* = 0.0354 and *P* = 0.0141, respectively). However, C2 took only 1.15 ± 0.05 h to complete the embryo rescue procedures, which was 2.43 times faster than C1 (2.80 ± 0.10 h; *P* < 0.0001). These results indicate that C1 is more accurate in identifying the embryo-containing caryopses, while C2 is substantially more efficient in completing the entire embryo rescue procedure, with only a slight embryo loss (1.4%), which also enables a remarkable reduction in the use of sterilants and labor in mass production of DH.

**Table 6:** Comparison of the efficiency and accuracy in embryo rescue between the routine method (C1) and its combination with pre-screening of caryopses using an optical device (C2)

Method	Accuracy (%)	False positive rate FPR (%)	False negative rate FNR (%)	Inspection time (h)
C1 (Mean ± SD)	100.0 ± 0.00	0.00 ± 0.00	0.00 ± 0.00	2.80 ± 0.10
C2 (Mean ± SD)	98.60 ± 0.20	0.47 ± 0.16	3.46 ± 0.72	1.15 ± 0.05
t-value	12.12	5.16	8.34	25.98
*P*-value	0.0067	0.0354	0.0141	< 0.0001
Significance	**	*	*	***

*Significance levels: *P < 0.05, **P < 0.01, ***P < 0.001

### Preliminary application of the improved protocol

To validate the practicality of this optimized protocol for the mass production of wheat DH. A three-year survey was conducted from 2022 to 2024, encompassing 463 spring, 315 facultative, and 261 winter wheat genotypes from 42 scientific research and breeding institutions across China, Bangladesh, Pakistan, Egypt, Germany, and CIMMYT. The results are summarized in Table 7. Over the three years, approximately 137,080 wheat DH lines were generated at YAAS, Kunming. On average, this protocol yielded 6.43 embryos per spike, a seedling formation rate of 66.47%, and a chromosome doubling rate of 64.74%. Compared with the 4,740 DH lines harvested in 2016 (unpublished data), approximately 50,820 DH lines were harvested in 2024, demonstrating the optimized protocol enables large-scale DH production, as evidenced by the three-year output.

**Table 7:** Summary of Doubled Haploid (DH) production efficiency across three consecutive years (2022–2024)

Season	Pollinated spikes	Embryo number	Haploid plantlets	Harvested DH lines	Embryos per spike (%)	Seedling formation rate (%)	Chromosome doubling rate (%)
2022	16,059	95,040	64,520	41,504	5.92	67.89	64.33
2023	16,410	103,770	67,850	44,756	6.32	65.38	65.96
2024	17,015	120,175	79,480	50,820	7.06	66.14	63.94
Total	49,484	318,985	211,850	137,080	-	-	-
Average	-	-	-	-	6.43	66.47	64.74

Embryos per spike is estimated as the number of embryos divided by the number of pollinated spikes; seedling formation rate is calculated as the percentage of haploid plantlets derived from the total number of cultured embryos; chromosome doubling rate means the percentage of doubled haploids relative to the number of colchicine-treated haploid plantlets

## Discussion

Doubled haploid technology using the wheat × maize system has been applied for three main purposes: (i) generating DH populations for genetic research [17, 42]; (ii) accelerating wheat breeding, especially in pyramiding multiple genes or desirable traits and population improvement [43–45]; (iii) purifying cultivars, sterile lines and restorers in hybrid wheat seed production [46]. Nevertheless, the widespread application of this DH technology, especially in wheat breeding, remains limited by the efficiency and cost of DH production [17, 39, 47]. Therefore, the improvements reported in this study enable more efficient production of wheat DH at a lower cost.

The efficiency and cost of DH production in wheat × maize system depend primarily on the way of how to achieve hybridization between wheat and maize [26, 48], technical stability, and continuous protocol optimization [8, 18, 47]. In some regions of South Asia and Africa, as well as some plateau regions around the world such as the Yunnan Plateau in China and the Mexican Plateau in North America, spring wheat and maize can be planted in the same season and their flowering periods overlap under natural conditions, which provides favorable conditions for the establishment and application of maize-mediated wheat DH technology, given the lower cost of repeatedly planting wheat and maize and virtually unlimited planting scale [29, 49]. However, the duration of the hybridization window between wheat and maize under natural conditions varies across regions, systematic research is therefore needed to evaluate the development and application potential of this DH technology [38]. Through the year-round sowing experiments of spring wheat and maize, this study determined that the hybridization window between wheat and maize under natural conditions in Kunming is five months (June to October), the same as that reported earlier [38], and can be further extended to 10 months (April to next January) by using low-cost high tunnels to plant maize from January to February and from August to September. For facultative and winter wheat, three strategies are feasible to produce DH at Kunming: (i) following vernalization, they can be planted year-round similar to spring wheat; (ii) without vernalization, they can be directly sown in early February; or (iii) these wheats are locally sown during the normal growing season to vernalize naturally, then transplanted to Kunming for wheat × maize hybridization, and before transplantation, candidate seedlings can be genotyped using marker-assisted selection, as exemplified in the recent report [45]. This study demonstrates that the Yunnan Plateau, particularly Kunming, offers unique and optimal natural conditions in China for the mass production of wheat DH via the wheat × maize system. It is supported by the observation that Yunnan has provided DH production services to over fifty institutions nationwide over the past five years (unpublished data). Moreover, 13 DH-derived cultivars were released in Yunnan Province from 2021 to 2025 [50], representing the highest number of DH-based cultivars released in China. Further research will focus on maize pollen supply during the period from February to March to enable year-round DH production. This will be achieved by growing maize in a glasshouse that is naturally heated using the abundant solar energy available during winter and spring in Kunming.

As alternatives, cogon grass (*Imperata cylindrica*) and gamagrass (*‌Tripsacum dactyloides*) can also serve as pollen donors to cross with wheat for DH production when maize pollen is unavailable [2, 15, 51]. However, their pollen production is far lower than that of maize, making them only suitable as supplementary strategies.

Pollinating wheat with maize pollen, floret-by-floret, is a fundamental yet labor-intensive and time-consuming task, even when a brush is used [3, 18, 52]. It also reduces pollen viability due to prolonged exposure [18, 40] when hundreds or thousands of wheat spikes require pollination. In this study, we developed a rolling pollination method that enables spike-by-spike pollination by leveraging the abundant fresh pollen from naturally and repeatedly grown maize. This transformative innovation in pollination method increased pollination efficiency by over tenfold, and raised the haploid formation frequency (EFF) by 1.9 times in this study, which may be attributed to two aspects: (i) minimal pollen viability loss due to short operation time, and (ii) complete stigma coverage maximizing fertilization opportunities. These factors need to be further investigated and confirmed. Additionally, maize pollen storage will also be explored in the next step.

Nutrient solution composition is a critical factor influencing haploid embryo induction during cut-tiller culture [28]. Based on previous reports [29, 49], we developed a further optimized nutrient solution composition that enabled a significant increase in the 1000-caryopsis weight and embryo formation frequency, which we attribute to the addition of boric acid (H_3_BO_3_) and citric acid (C_6_H_8_O_7_), combined with the removal of sulfurous acid (H_2_SO_3_) and silver nitrate (AgNO_3_) from the currently used solution. Boron is known to play an essential role in pollen tube growth, fertilization, and early seed development by stabilizing cell wall structure and regulating carbohydrate metabolism [53, 54]. In wheat × maize system, sufficient boron supply may enhance maize pollen germination, pollen tube penetration, and fertilization with wheat, thereby increasing caryopsis set and embryo formation.Citric acid, a chelating agent and antioxidant, helps maintain solution stability and prevents oxidative damage to the developing caryopses [55]. The removal of sulfurous acid and silver nitrate not only reduced potential phytotoxicity but also addressed environmental concerns. Visual differences in caryopsis appearance–plump and translucent under solution A versus shriveled and pale under solution B–further support the physiological benefits of solution A. Results of this study are consistent with previous reports that optimized nutrient solutions improve haploid induction in detached tiller culture [2, 29, 56, 57].

Compared with routine embryo rescue procedures [18, 21, 22, 26] and the light-based caryopsis detection technique [58], pre-screening embryo-containing caryopses using a newly designed optical device prior to rescue increased operating efficiency to 2.43 times that of the conventional method, with only a 1.4% embryo loss rate and a 3.46% false negative rate in our study. Using transmitted light with dual magnification (lens + mirror) in the device makes the embryo directly visible regardless of its position within the caryopsis. This overcomes a key limitation of the method reported earlier [58], which relied on the embryo settling at the bottom of the seed, an assumption not consistently met in our large-scale practice. Therefore the application of the new device not only doubles the efficiency of embryo rescue but also enables a remarkable reduction in the use of sterilants and labor in mass production of DH, where tens of thousands of caryopses need to be processed daily.

The optimized protocols in this study enhanced DH production efficiency and reduced its cost. and were verified by the DH production from 2022 to 2024, yielding over 137,080 DH lines with 1,039 diverse wheat materials from more than 40 institutions (unpublished data). It is expected that the optimized protocol for wheat DH production will be applicable to regions with similar climates to accelerate wheat breeding and enhance food security.

## Conclusions

This study further optimized the wheat × maize based DH production protocol in four aspects: (i) clarified the annual crossing window for wheat × maize at Kunming is five months (June to October) under field conditions, and can be further extended to ten months (April to next January) by using high-tunnel cultivation of maize from January to February and from August to September; (ii) rolling pollination, a transformative pollination method, enables to increase pollination efficiency by over tenfold, and to raise the haploid formation frequency by 1.9 times; (iii) optimized nutrient solution exhibits reduced cost, enhanced embryo induction efficiency and improved environmental performance; (iv) pre-screening embryo-containing caryopses using a newly-designed optical device prior to embryo rescue, had a 98.6% accuracy and a 3.46% false negative rate in selecting embryo-containing caryopses. This method not only doubles the efficiency of embryo rescue but also enables a remarkable reduction in the use of sterilants and labor in the mass production of DH. The optimized protocols significantly enhanced DH production efficiency and reduced its costs, which were verified from 2022 to 2024, yielding over 137,080 DH lines from 1,039 diverse wheat materials. It is expected that the optimized protocol for wheat DH production will be applicable to regions with similar climates.

## Supplementary Information

Supplementary Material 1.

## Data Availability

All data and materials generated or analyzed during this study are included in this article or are available from the corresponding author on reasonable request.

## Abbreviations

YAAS: Yunnan Academy of Agricultural Sciences
DH: Doubled haploid
EFF: Embryo formation frequency
DTH: Days to heading
RCBD: Randomized complete block design
PE: Pollination efficiency
CSR: Caryopsis setting rate
SR: Selfing rate
CW: 1000-caryopses weight
C1 and C2: Inspection methods
FPR: False positive rate
FNR: False negative rate
TP: True positive
TN: True negative
FP: False positive
FN: False negative
PE: Pollination efficiency
FLSD: Fisher’s least significant difference
