# Supplementary material for: Leveraging natural climatic advantages for large‑scale wheat doubled haploid production via wheat × maize: a protocol optimization study
Source: BMC Plant Biol. 2026 May 26;26:1349. doi: 10.1186/s12870-026-09049-w (PMC13459365; doi:10.1186/s12870-026-09049-w)
Supplement: Supplementary file 1 [file 12870_2026_9049_MOESM1_ESM.docx]

**Table S1.** Summary of days to heading (DTH) for Fifty-two sowing dates (SD) of seven spring bread wheat genotypes.

| **Code** | **Sowing Date** | **Min.** | **Max.** | **Mean** | **Std. Error** | **Code** | **Sowing Date** | **Mini.** | **Max.** | **Mean** | **Std. Error** |
| --- | --- | --- | --- | --- | --- | --- | --- | --- | --- | --- | --- |
| SD01 | 1-Jan | 100 | 103 | 101 | 0.5 | SD27 | 2-Jul | 52 | 59 | 56 | 0.9 |
| SD02 | 8-Jan | 93 | 100 | 96 | 0.9 | SD28 | 9-Jul | 56 | 65 | 62 | 1.3 |
| SD03 | 15-Jan | 88 | 95 | 90 | 1.0 | SD29 | 16-Jul | 60 | 69 | 65 | 1.0 |
| SD04 | 22-Jan | 84 | 93 | 87 | 1.2 | SD30 | 23-Jul | 56 | 64 | 61 | 1.1 |
| SD05 | 29-Jan | 80 | 88 | 83 | 1.2 | SD31 | 30-Jul | 59 | 65 | 64 | 0.9 |
| SD06 | 5-Feb | 76 | 81 | 79 | 0.9 | SD32 | 6-Aug | 58 | 70 | 66 | 1.8 |
| SD07 | 12-Feb | 71 | 76 | 73 | 0.9 | SD33 | 13-Aug | 61 | 73 | 70 | 1.6 |
| SD08 | 19-Feb | 65 | 71 | 68 | 0.9 | SD34 | 20-Aug | 69 | 76 | 73 | 1.0 |
| SD09 | 26-Feb | 64 | 66 | 65 | 0.4 | SD35 | 27-Aug | 65 | 83 | 77 | 2.1 |
| SD10 | 5-Mar | 60 | 64 | 62 | 0.6 | SD36 | 3-Sep | 78 | 88 | 80 | 1.3 |
| SD11 | 12-Mar | 57 | 62 | 59 | 0.8 | SD37 | 10-Sep | 70 | 97 | 79 | 3.1 |
| SD12 | 19-Mar | 54 | 60 | 57 | 0.9 | SD38 | 17-Sep | 73 | 113 | 96 | 5.0 |
| SD13 | 26-Mar | 52 | 60 | 56 | 1.2 | SD39 | 24-Sep | 102 | 131 | 114 | 3.6 |
| SD14 | 2-Apr | 48 | 56 | 52 | 1.1 | SD40 | 1-Oct | 101 | 128 | 120 | 3.4 |
| SD15 | 9-Apr | 51 | 56 | 54 | 0.7 | SD41 | 8-Oct | 114 | 137 | 125 | 3.0 |
| SD16 | 16-Apr | 50 | 58 | 55 | 1.1 | SD42 | 15-Oct | 124 | 134 | 129 | 1.6 |
| SD17 | 23-Apr | 51 | 56 | 55 | 0.8 | SD43 | 22-Oct | 125 | 134 | 129 | 1.3 |
| SD18 | 30-Apr | 50 | 58 | 54 | 1.3 | SD44 | 29-Oct | 122 | 134 | 128 | 1.6 |
| SD19 | 7-May | 52 | 61 | 58 | 1.3 | SD45 | 5-Nov | 119 | 133 | 128 | 1.9 |
| SD20 | 14-May | 50 | 61 | 57 | 1.3 | SD46 | 12-Nov | 113 | 126 | 120 | 1.7 |
| SD21 | 21-May | 56 | 67 | 60 | 1.3 | SD47 | 19-Nov | 119 | 123 | 122 | 0.6 |
| SD22 | 28-May | 53 | 62 | 57 | 1.2 | SD48 | 26-Nov | 115 | 121 | 118 | 0.7 |
| SD23 | 4-Jun | 52 | 61 | 57 | 1.2 | SD49 | 3-Dec | 87 | 117 | 112 | 4.2 |
| SD24 | 11-Jun | 55 | 60 | 58 | 0.6 | SD50 | 10-Dec | 109 | 115 | 111 | 0.8 |
| SD25 | 18-Jun | 54 | 58 | 57 | 0.7 | SD51 | 17-Dec | 105 | 109 | 107 | 0.5 |
| SD26 | 25-Jun | 54 | 60 | 58 | 0.8 | SD52 | 24-Dec | 101 | 107 | 104 | 0.8 |
| Probability of sowing dates = 0.001  Probability of genotypes = 0.893 | | | | | | | | | | | |

**Table S2.** Sowing and flowering dates of maize under field and high tunnel in 2020 and 2021.

| **Field environment** | | | | **High tunnel environment** | | | |
| --- | --- | --- | --- | --- | --- | --- | --- |
| **Sowing Date (2020)** | **Flowering Date**  **(2020)** | **Sowing Date (2021)** | **Flowering Date**  **(2021)** | **Sowing Date (2020)** | **Flowering Date**  **(2020)** | **Sowing Date (2021)** | **Flowering Date**  **(2021)** |
| 4-Mar | 4-Jun | 1-Mar | 5-Jun | 14-Jan | 16-Apr | 1-Jan | 4-Apr |
| 18-Mar | 18-Jun | 15-Mar | 20-Jun | 28-Jan | 30-Apr | 15-Jan | 2-May |
| 1-Apr | 3-Jul | 29-Mar | 4-Jul | 9-Feb | 10-May | 29-Jan | 10-May |
| 15-Apr | 14-Jul | 12-Apr | 14-Jul | 23-Feb | 27-May | 12-Feb | 21-May |
| 29-Apr | 26-Jul | 26-Apr | 23-Jul | 5-Aug | 12-Nov | 1-Aug | 14-Nov |
| 13-May | 10-Aug | 10-May | 5-Aug | 19-Aug | 25-Nov | 15-Aug | 27-Nov |
| 27-May | 25-Aug | 24-May | 20-Aug | 2-Sep | 16-Dec | 29-Aug | 17-Dec |
| 10-Jun | 6-Sep | 7-Jun | 2-Sep | 16-Sep | 30-Dec | 12-Sep | 23-Dec |
| 24-Jun | 20-Sep | 21-Jun | 17-Sep | 30-Sep | 10-Jan | 26-Sep | 8-Jan |
| 8-Jul | 11-Oct | 5-Jul | 3-Oct |  |  |  |  |
| 22-Jul | 23-Oct | 19-Jul | 15-Oct |  |  |  |  |
